# Phylogenetic variation in raw cow milk microbiota and the impact of forage combinations and use of silage inoculants

**DOI:** 10.3389/fmicb.2023.1175663

**Published:** 2023-11-07

**Authors:** Alexandre J. K. Ouamba, Mérilie Gagnon, Thibault Varin, P. Yvan Chouinard, Gisèle LaPointe, Denis Roy

**Affiliations:** ^1^Département des Sciences des Aliments, Laboratoire de Génomique Microbienne, Université Laval, Québec, QC, Canada; ^2^Regroupement de Recherche pour Un Lait de Qualité Optimale (Op^+^Lait), Saint-Hyacinthe, QC, Canada; ^3^Département des Sciences Animales, Université Laval, Québec, QC, Canada; ^4^Department of Food Science, University of Guelph, Guelph, ON, Canada

**Keywords:** raw milk microbiota, forage ration, viable bacteria, qPCR, silage microbial additives, bacterial transfer, dairy farm

## Abstract

**Introduction:**

The microbiota of bulk tank raw milk is known to be closely related to that of microbial niches of the on-farm environment. Preserved forage types are partof this ecosystem and previous studies have shown variations in their microbial ecology. However, little is known of the microbiota of forage ration combinations and the transfer rates of associated species to milk.

**Methods:**

We identified raw milk bacteria that may originate from forage rations encompassing either hay (H) or grass/legume silage uninoculated (GL) as the only forage type, or a combination of GL and corn silage uninoculated (GLC), or grass/legume and corn silage both inoculated (GLICI). Forage and milk samples collected in the fall and spring from 24 dairy farms were analyzed using 16S rRNA gene high-throughput sequencing following a treatment with propidium monoazide to account for viable cells.

**Results and discussion:**

Three community types separating H, GL, and GLICI forage were identified. While the H community was co-dominated by *Enterobacteriaceae*, *Microbacteriaceae*, *Beijerinckiaceae*, and *Sphingomonadaceae*, the GL and GLICI communities showed high proportions of *Leuconostocaceae* and *Acetobacteraceae*, respectively. Most of the GLC and GLICI rations were similar, suggesting that in the mixed forage rations involving grass/legume and corn silage, the addition of inoculant in one or both types of feed does not considerably change the microbiota. Raw milk samples were not grouped in the same way, as the GLC milk was phylogenetically different from that of GLICI across sampling periods. Raw milk communities, including the GLICI group for which cows were fed inoculated forage, were differentiated by *Enterobacteriaceae* and other Proteobacteria, instead of by lactic acid bacteria. Of the 113 amplicon sequence variants (ASVs) shared between forage rations and corresponding raw milk, bacterial transfer rates were estimated at 18 to 31%. Silage-based forage rations, particularly those including corn, share more ASVs with raw milk produced on corresponding farms compared to that observed in the milk from cows fed hay. These results show the relevance of cow forage rations as sources of bacteria that contaminate milk and serve to advance our knowledge of on-farm raw milk contamination.

## 1. Introduction

The microbiological quality of raw milk is essential for its safety and processability. On dairy farms, the complex community of raw milk (Quigley et al., 2013) gradually builds up as the milk is collected from the mammary gland of the cow (Porcellato et al., 2020; Rault et al., 2020) through the teat canal and the milking equipment (Verdier-Metz et al., 2012; Falentin et al., 2016) to a cooled bulk tank. Factors inherent to the cow such as the health status of the udder (Oikonomou et al., 2014; Derakhshani et al., 2018a) or the lactation stage (Doyle et al., 2017a; McHugh et al., 2020), or of environmental origin including air, pasture, feces, bedding, teat surface, water, and feed (Gleeson et al., 2013; Quigley et al., 2013; Derakhshani et al., 2018b; Gagnon et al., 2020a) as well as management practices (Ouamba et al., 2022), have been found to influence the occurrence of microorganisms in raw milk. The impact of seasons and weather conditions on changes in the milk microbiota throughout the year has been demonstrated (Kable et al., 2016; Doyle et al., 2017b; Li et al., 2018; Bettera et al., 2023). Milk refrigeration upon milking as recommended favors the proliferation of psychrotrophic bacteria (Rasolofo et al., 2010; Vithanage et al., 2016; Kennang Ouamba et al., 2020; Salgado et al., 2020), and also mesophiles including lactococci, enterococci, streptococci, or lactobacilli that can withstand temperatures as low as 4°C (Rasolofo et al., 2010; Vithanage et al., 2016; Hahne et al., 2019; Kennang Ouamba et al., 2020).

Management practices implemented to maintain dairy farm profitability and improve the quality of products encompass a variety of measures among which housing, use of antibiotics, milking routine, bedding, cow hygiene, and herd nutrition are the most important of those associated with changes in the raw milk microbiota (Doyle et al., 2017a; Derakhshani et al., 2018a; Murphy et al., 2019). Dry and ensiled grass or legumes, which can be supplemented with corn silage, constitute the most common feed components for dairy cows (Hassanat et al., 2013). In a previous study (Kennang Ouamba et al., 2022), we used high-throughput sequencing of the 16S rRNA gene to compare the composition and structure of the bacterial community populating farm-scale produced hay and grass/legume or corn silage, among which the last two were ensiled with or without inoculants. We found, as have others (Ni et al., 2017; Ávila and Carvalho, 2020; Daniels et al., 2020) that these distinct forage types also harbored phylogenetically different community assemblies which included, besides technologically relevant bacteria (Colombo et al., 2018), pathogenic and spoilage microorganisms that can contaminate raw milk and cause serious defects during milk processing (Driehuis, 2013; Driehuis et al., 2018). Doyle et al. (2017b) previously compared the sources of raw milk contamination on the farm, and found that grass silage was a minor contributor, after teat surface and feces, to the microbiota of bulk tank milk produced by cows housed indoors. However, our knowledge of the prevalence and diversity of raw milk microbial species that originate from forage types including hay and grass/legume or corn silage is limited. Ensiling most often requires the addition of commercial inoculants to ensure high quality silage with enhanced aerobic stability and nutritional value. Inoculants are mainly composed of homofermentative or facultative heterofermentative lactic acid bacteria (LAB) such as *Lactiplantibacillus plantarum*, *Lacticaseibacillus casei*, and *Pediococcus* spp., obligate heterofermentative LAB such as *Lentilactobacillus buchneri* and *Lentilactobacillus hilgardii*, or combination of these species. Despite the increasing interest in silage inoculants with improved fermentative capabilities and high potential for silage aerobic stability and animal productivity (Wilkinson and Muck, 2019; Drouin et al., 2020; Guan et al., 2020; Nair et al., 2020), little is known about the impact of these commercial inoculants on the raw milk microbiota and processability. Moreover, there is a lack of knowledge of the patterns of raw milk contamination on farm that are driven by silage management practices. The main objective of this study was to investigate the impact of feeding dairy cows with dry or ensiled forage, whether inoculated or uninoculated, on raw milk microbiota.

## 2. Materials and methods

### 2.1. Forage and milk sampling

The forage types and raw milk samples were collected from 24 dairy farms in fall 2015 and spring 2016. The culture-dependent analysis of the milk and forage is described in Gagnon et al. (2020b) while the 16S rRNA gene profiles of the microbial community of individual forage types is described in Kennang Ouamba et al. (2022). For forage, sampling and sample treatments were processed in the same way as previously described (Kennang Ouamba et al., 2022). For milk, the distinct processing steps required for DNA extraction were performed as previously described (Kennang Ouamba et al., 2020). Briefly, the 24 dairy farms implemented five cow feeding practices encompassing either hay (H) or grass/legume silage uninoculated (GL) as the sole forage types, or a combination of GL and corn silage uninoculated (GLC) or inoculated (GLCI), or grass/legume and corn silage both inoculated (GLICI). Proportions of corn silage in forage combinations ranged between 38 and 74% of the total mixture obtained. Forage rations therefore included H, GL, GLC, GLCI, and GLICI feeding combinations counting 5, 7, 4, 1, and 7 herds, respectively, for each of the two sampling periods. Commercial inoculants used for ensiling included Biotal Buchneri 500 and Biotal Supersile from Lallemand Animal Nutrition (Milwaukee, WI), and 11C33, 11CFT, and 11G22 from Pioneer (Johnston, IA). Raw milk samples collected from bulk tanks (100 mL) were conveyed refrigerated to the laboratory as described previously (Kennang Ouamba et al., 2020; Gagnon et al., 2020b). Pellets obtained from 10 mL aliquots of raw milk samples were treated with propidium monoazide (PMA) as previously described (Kennang Ouamba et al., 2020) to account for viable cells. PMA-treated pellets were then stored at −80°C until DNA extraction.

### 2.2. DNA extraction, sequencing, and PCR quantification

Genomic DNA extraction from forage and milk samples was performed using the DNeasy PowerFood Microbial Kit (Qiagen, Hilden, Germany) following enzymatic lysis with mutanolysin from *Streptomyces* (MilliporeSigma), lysozyme (MilliporeSigma), and proteinase K (MilliporeSigma) as previously described (Kennang Ouamba et al., 2020). Genomic DNA samples were sent for sequencing at the *Plateforme d’Analyses Génomiques* of *Université Laval* (Quebec, Canada). The V3-V4 region of the 16S rRNA gene was amplified using the 347F (5′-GGAGGCAGCAGTRRGGAAT) and 803R (5′-CTACCRGGGTATCTAATCC) primers. Using specific primer sets as described previously (Kennang Ouamba et al., 2022), milk loads of *Lpb. plantarum*, *Len. buchneri*, total lactic acid bacteria (LAB), total acetic acid bacteria (AAB), *Pseudomonas*, *Enterobacteriaceae*, total bacteria, and total fungi were determined by qPCR. These bacterial species or groups were chosen based on their abundance in silage and raw milk from previous studies (Kennang Ouamba et al., 2020, 2022). Results were expressed as log copy numbers per milliliter of milk.

### 2.3. Bioinformatic and data analyses

Raw sequences were quality checked and processed using the software FastQC (Version 0.11.9), Cutadapt (version 2.3), and the DADA2 pipeline (Martin, 2011; Callahan et al., 2016) as previously described (Kennang Ouamba et al., 2020). The same forage sequences analyzed previously (Kennang Ouamba et al., 2022) were used in this study. However, a new dataset was formed using the five categories of the feeding combinations described above by merging (1/1 ratio of read counts) the sequence data associated with forage samples. As the GLCI forage combination consisted of only one sample per sampling period, the corresponding data, as well as those from the associated raw milk, were removed from the dataset. Although forage sequences were examined separately from those of milk, their corresponding sequence tables were merged using the function *mergeSequenceTables()* before removing chimeras and assigning taxonomy as described in the DADA2 tutorial for big data. The Silva release 132 was used for taxonomy assignment. Forage and milk processed sequences were therefore assigned ASVs (amplicon sequence variant) names at the same time, and downstream analyses regarding alpha and beta diversity were performed using the phyloseq (version 1.30.0) package (McMurdie and Holmes, 2013) on the PMA-treated dataset.

For the alpha-diversity analysis, Chao1, Shannon, and Inverse Simpson indices were computed. Local contribution to beta diversity (LCBD) and principal coordinate or component analyses were used to capture the beta-diversity (Ssekagiri et al., 2020). Statistical analysis of group comparisons was performed on sequence data transformed by Centered Log-Ratio (CLR) or by Phylogenetic Isometric Log-Ratio (PhILR) to assess the compositional and the phylogenetic structures of the milk microbiota (Gloor et al., 2017; Silverman et al., 2017). Kruskal-Wallis and post-hoc tests with false discovery rate correction of the *p*-values were performed. The R package ALDEx2 4.0 was used to compute taxa differential abundance (Fernandes et al., 2014). The R package ComplexHeatmap 2.4.2 (Gu et al., 2016) was used for data visualization.

The PhILR transformed milk dataset was analyzed using a sparse logistic regression model implemented in the glmnet (version 3.0.2) R package (Friedman et al., 2010) to identify “balances” (log-ratios of the geometric mean relative abundances of adjacent clades) that discriminate milk samples between forage combinations (Silverman et al., 2017).

Silage and milk data were clustered using the partitioning around medoids (PAM) algorithm on Euclidean distances calculated from PhILR transformed data using the factoextra 1.0.7 (Kassambara and Mundt, 2017) R package. In addition to silhouette analysis, ordination in the PhILR space was performed as previously described to validate the cluster analysis results. Forage rations were then classified into different forage ration community types defined by the number of clusters obtained. Milk samples corresponding to forage rations were analyzed the same way.

Co-occurring ASVs among forage types composing each forage combination and the associated milk samples were investigated by calculating intersects with the software VENN DIAGRAMS available online at http://bioinformatics.psb.ugent.be/webtools/Venn/. The abundance and distribution of shared ASVs among forage and corresponding milk in each combination were visualized by constructing a heatmap and a chord diagram using Complexheatmap (Gu et al., 2016) and Circos v0.63–9 (Krzywinski et al., 2009), respectively.

## 3. Results

### 3.1. Microbial community types of forage combinations

**Figure 1:**
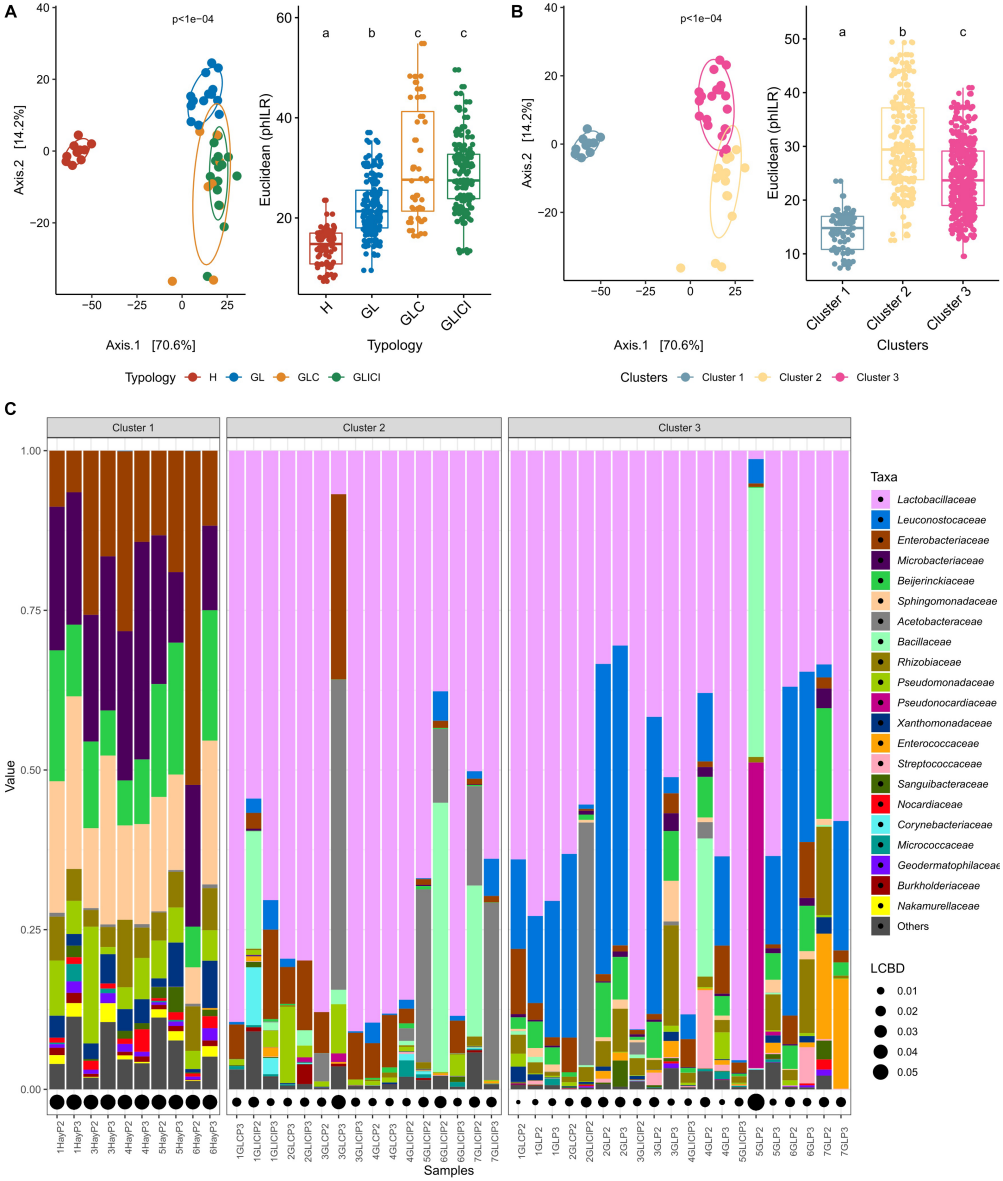
Forage ration community types. Principal component analysis based on PhILR transformed data (left) with corresponding post-hoc tests (right) for forage ration types **(A**) and derived clusters **(B)**. Forage feeding typologies or clusters associated with different lower-case letters are significantly different based on Wilcoxon rank sum test with FDR correction. **(C)** Relative abundance of the 21 most abundant families occurring in the defined clusters. Local contribution to beta diversity values denotes the indices of sample local contribution to the observed beta-diversity between groups. Values are proportional to sample contribution to beta-diversity. For more results on cluster analysis, see Supplementary Figure S1 in the Supplementary material.

Significant differences in the phylogenetic structure of microbial communities were found between H and GL, GLC or GLICI, as well as between those of GL and GLC or GLICI forage combinations (Figure 1A). The bacterial communities provided by GLC and GLICI forage rations were phylogenetically similar. Cluster analysis of forage rations at the farm level showed three community types (Figure 1B, Supplementary Figure S1A). Cluster 1 gathered all samples of the H type, broadly exhibiting co-dominance of *Enterobacteriaceae*, *Microbacteriaceae*, *Sphingomonadaceae*, *Beijerinckiaceae*, and *Pseudomonadaceae* (Figure 1C). Most GLICI forage rations composed cluster 2. Samples in this cluster were either largely dominated by *Lactobacillaceae* or co-dominated by *Lactobacillaceae* and *Acetobacteraceae* or *Bacillaceae*. Cluster 3 gathered almost all GL samples, characterized by relatively greater proportions of *Beijerinckiaceae*, *Rhizobiaceae*, or *Enterococcaceae*. Additionally, forage rations in cluster 3 were either largely dominated by *Lactobacillaceae* or co-dominated by *Lactobacillaceae* and *Leuconostocaceae*. Forage rations co-dominated by *Acetobacteraceae* and *Enterobacteriaceae* (e.g., sample 3GLP3) or by *Bacillaceae* and *Pseudonocardiaceae* (e.g., sample 5GLP2) exhibited greater LCBD indices compared with others within clusters 2 and 3, respectively. The same pattern of variations in the phylogenetic structure (Supplementary Figures S1B, S2A) of the microbiota revealed for forage rations was also observed among raw milk samples. However, although the cluster analysis of raw milk samples showed three clusters (Supplementary Figure S2B), they were not grouped in the same way as for forage rations. Of the three clusters obtained for milk, two gathered 91% of samples, mostly separated by sampling period (Supplementary Figure S2C).

### 3.2. Diversity of raw milk microbial communities

**Figure 2:**
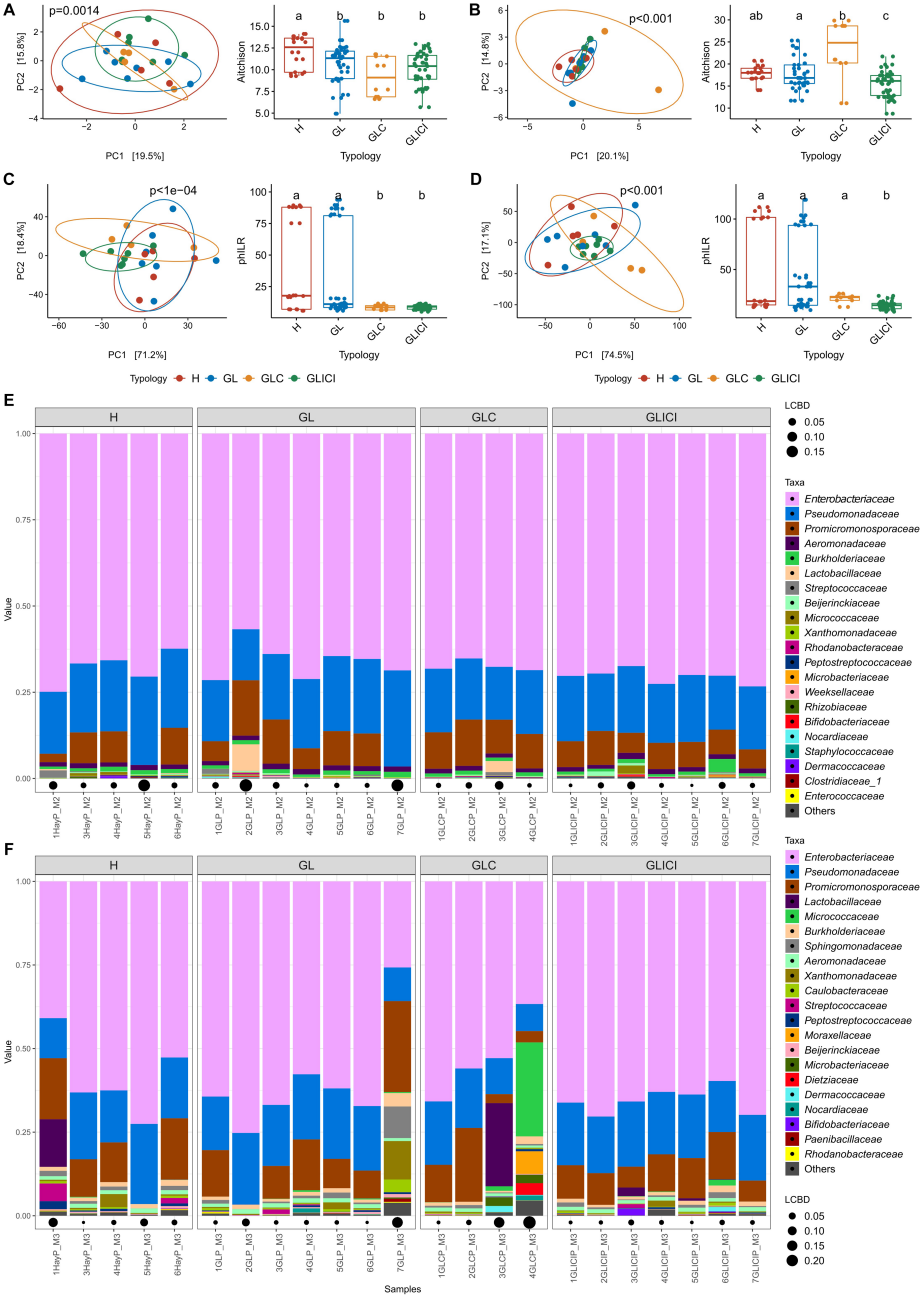
Milk community diversity and composition across forage types. Principal component analysis (left) with corresponding post-hoc test (right) based on CLR transformed data of milk samples in the fall **(A)** and the spring **(B)** or based on PhILR transformed data in the fall **(C)** and the spring **(D)**. Milk groups associated with different letters are significantly different based on Wilcoxon rank sum test with FDR correction. Relative abundance of the 21 most abundant genera in the microbiota of milk samples collected in the fall **(E)** and the spring **(F)**. Local contribution to beta diversity values denotes the indices of sample local contribution to the observed beta-diversity between groups. Values are proportional to sample contribution to the beta diversity. Different lower-case letters represent statistically significant differences between milk groups.

Similar alpha-diversity metrics (Chao1, Shannon, and inverse Simpson indices) were observed between raw milk samples across forage ration combinations (Supplementary Figure S3). Beta-diversity analysis showed that in the fall, GL, GLC, and GLICI milk samples harbored similar microbial community structures, each significantly different from that of H milk (Figure 2A). In the spring, while H milk samples showed similar community structures with GL and GLC, a significant difference was observed between H and GLICI (Figure 2B). Interestingly, GL, GLC and GLICI milk samples exhibited significantly different community structures (*p* < 0.05) from each other. However, in the fall, milk microbial communities of H compared with GL, or GLC versus GLICI were phylogenetically similar, while those of H and GL were significantly different from GLC and GLICI (Figure 2C). In the spring, H, GL, and GLC milk samples were phylogenetically similar, but each was significantly different from GLICI (Figure 2D). Regardless of the sampling period, *Enterobacteriaceae* and *Pseudomonadaceae* generally dominated the microbiota of milk samples. In addition to *Enterobacteriaceae* and *Pseudomonadaceae*, samples exhibiting higher LCBD indices were enriched in either one or combinations of the families *Promicromonosporaceae*, *Lactobacillaceae*, *Micrococcaceae*, *Streptococcaceae*, *Sphingomonadaceae*, *Xanthomonadaceae*, *Moraxellaceae*, and *Dietziaceae* (Figures 2E,F).

Almost all the ASVs found to be differentially abundant between milk samples in the fall and spring (96% of 47 ASVs) were Proteobacteria, and the remaining were classified as Actinobacteria (Supplementary Figures S4A,B). Among these taxa, *Pseudomonas*, unclassified *Enterobacteriaceae, Serratia*, and *Cellulosimicrobium* were the most abundant. Compared with GL or GLC, most of the differentially abundant taxa exhibited greater relative abundance in GLICI milk samples across both sampling periods.

We identified 19 bacterial clade ratios called balances (see the Materials and methods section for details) that discriminated between milk samples associated with the forage types (Supplementary Figure S5). Among balances, most Proteobacteria were involved at all taxonomic levels. The abundance of Firmicutes relative to Actinobacteria distinguished GLICI or GLC from GL or H milk samples.

**Figure 3:**
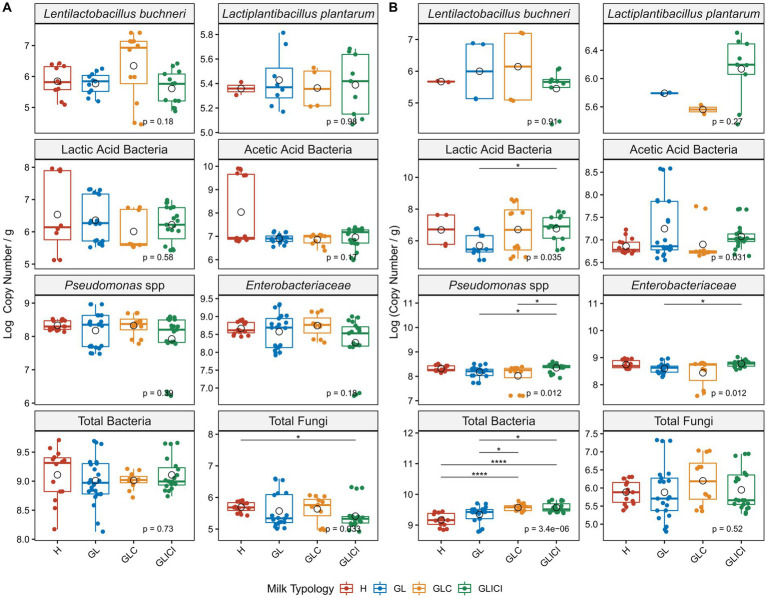
Comparative analysis of milk viable microbial loads across forage types. Microbial loads expressed in log copy numbers are compared between H milk sample and those from GL, GLC, and GLICI, or between GLICI milk samples and those from GLC or GL in the fall **(A)** and the spring **(B)**. Circle open shape within the box indicates the mean value for each group. *p* values are obtained following a Kruskal Wallis test. Asterisks above boxes indicate significant differences and flag *p*-values from a Wilcoxon rank test as follows: *, *p* < 0.05; **, *p* < 0.01; ***, *p* < 0.001; ****, *p* < 0.0001.

Quantitative PCR analyses performed to estimate viable microbial loads in milk revealed that neither *Len. buchneri*, *Lpb. plantarum*, LAB, AAB, *Pseudomonas* spp., *Enterobacteriaceae*, nor total bacteria varied significantly between milk samples in the fall (Figure 3A). However, a significantly lower abundance (*p* < 0.05) of total fungi was observed in GLICI compared with H milk samples (Figure 3A). In the spring, a significant enrichment of LAB was observed in GLICI compared with GL milk samples (*p* < 0.05), as were *Pseudomonas* in GLICI compared with GLC and GL milk samples, respectively (*p* < 0.05), and *Enterobacteriaceae* in GLICI compared with GL milk samples (*p* < 0.05). Total bacterial loads were significantly greater in GLICI or GLC compared with GL (*p* < 0.05) and H (*p* < 0.0001), respectively (Figure 3B). Although not significant, *Len. buchneri* and *Lpb. plantarum* levels were consistently greater in GLC and GLICI milk samples, respectively, across both sampling periods.

### 3.3. Distribution of *Lactobacillales* in raw milk

**Figure 4:**
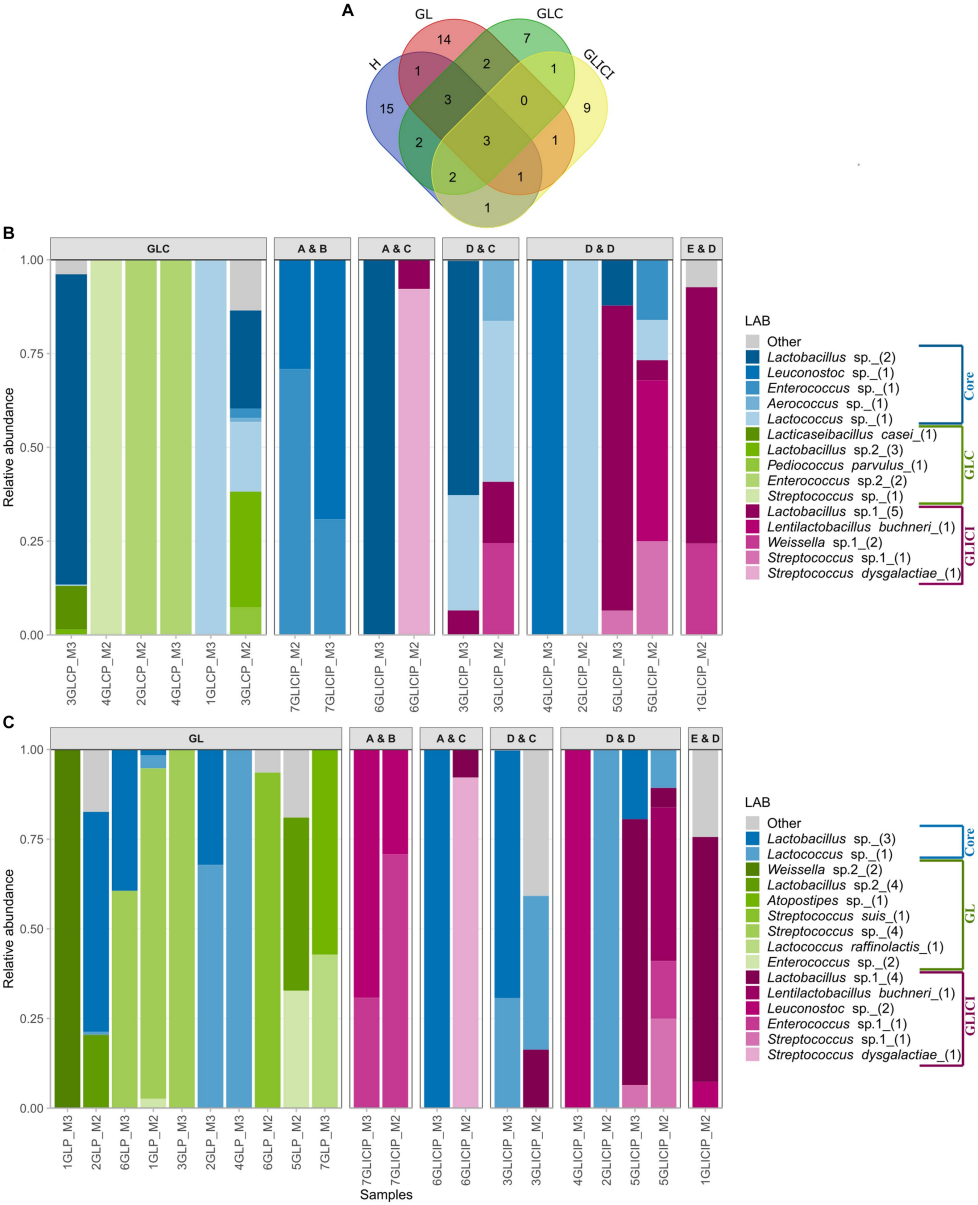
Distribution and proportion of lactic acid bacteria (LAB) among milk groups. **(A)** Venn diagram of LAB ASVs among milk groups. Proportion of the core and unique LAB ASVs among GLICI and GLC **(B)** or GL **(C)** milk samples. Milk samples originating from inoculated forage types are separated according to the inoculants used for the grass or legume (first letter) and corn (second letter) silage. Accordingly, “A” = 11G22, “B” = 11C33, “C” = 11CFT, “D” = Biotal Buchneri 500, and “E” = Biotal Supersile. Taxa are colored according to their unicity to a group (GLC versus GLICI, GLICI versus GL) or whether they were shared (core) by group pairs.

The raw milk dataset was comprised of 569 ASVs of which those assigned to the order *Lactobacillales* (~11%) accounted for only 1% of total relative abundance. Broadly, 28 LAB ASVs occurred in H milk samples, 25 in GL, 20 in GLC, and 18 in GLICI milk samples (Figure 4A). The core LAB among milk samples included *Lactobacillus* represented by two ASVs, and *Lactococcus* represented by a single ASV. Within each feeding combination, none of the core LAB ASVs, those shared between group pairs, nor those found specific to a group (Figures 4B,C; Supplementary Figures S6A,B) were consistently detected among milk samples. Moreover, several samples harbored one or two ASVs of either *Lactococcus*, *Lactobacillus*, *Leuconostoc*, *Streptococcus*, *Weissella*, or *Enterococcus* as the sole representatives of the LAB community.

### 3.4. Shared bacteria between forage types and the corresponding raw milk microbiota

**Figure 5:**
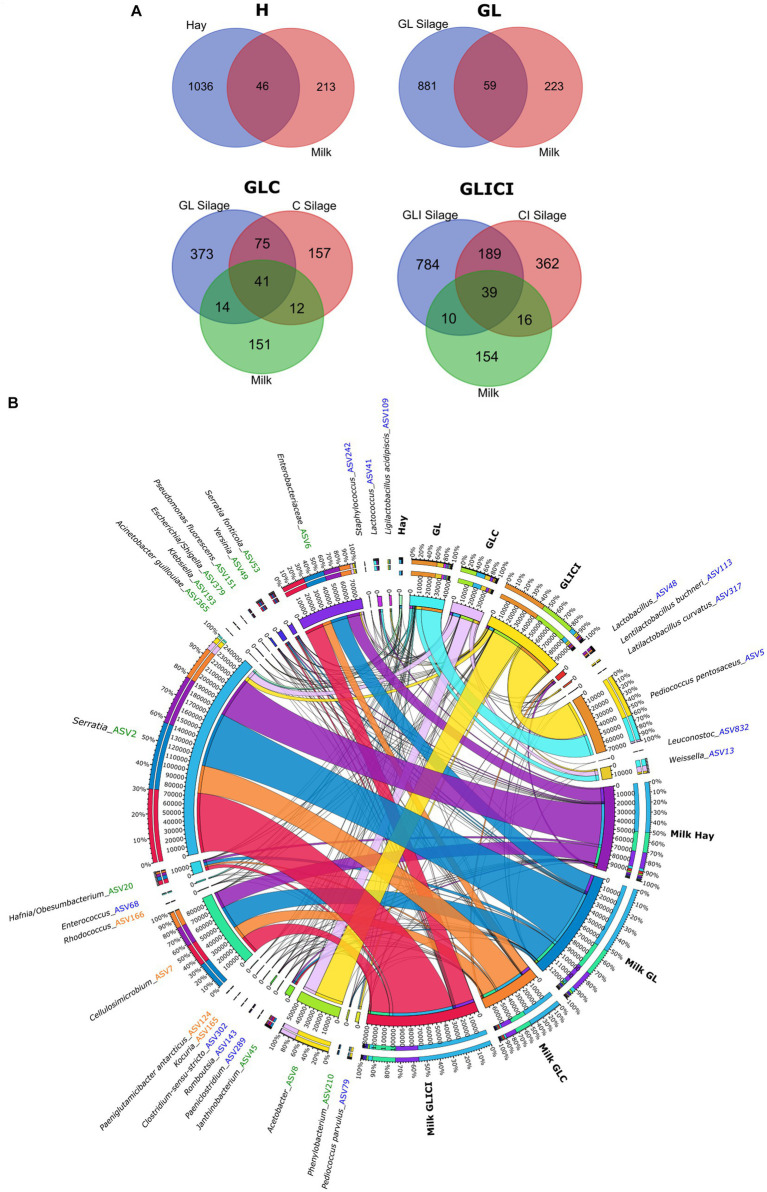
The core ASVs between forage types and corresponding milk samples. **(A)** Venn diagrams showing the number of ASVs shared between forage types and corresponding milk samples. **(B)** Chord diagram illustrating the distribution of the 30 more abundant ASVs (out of 113) potentially transferred from forage types to milk. For the full list of taxa potentially transferred, see Supplementary Figure S7 in the Supplementary material.

Comparing bacterial communities between preserved forage and associated raw milk within the same feeding combination, we identified common ASVs between both ecosystems. The proportions of shared and unique ASVs between forage and milk varied across feeding typologies (Figure 5A). Since each ASV is unique in the whole dataset and given that the bulk tank raw milk microbiota originates from the dairy farm and its vicinity, we assumed that the concurrent occurrence of an ASV in both forage and raw milk was plausibly the consequence of a transfer from forage to milk, with no assumption on the mode of transfer. Lower bacterial transfer rates from forage to milk were observed in H and GL feeding combinations, where milk samples shared 18 and 21% of their microbial community with corresponding forage rations, respectively. Greater bacterial transfer rates from forage rations to milk were observed for GLC and GLICI forage combinations, as milk samples shared 31 and 30% of their bacterial ASVs with the respective associated forage.

A total of 113 ASVs shared between forage and milk were identified, encompassing 74 assigned to Proteobacteria, 26 to Firmicutes, and 13 to Actinobacteria (Figure 5B, Supplementary Figure S7). Proteobacteria including ASVs of *Serratia* and an unclassified *Enterobacteriaceae*, and Actinobacteria including *Cellulosimicrobium* largely dominated the milk microbial community considered to originate from forage. However, Firmicutes included, but were not limited to, ASVs of *Lactobacillus*, *P. pentosaceus*, *Len. buchneri*, *Weissella*, *Lactococcus*, *Enterococcus*, *Leuconostoc*, *Clostridium*, and *Staphylococcus*. Interestingly, although these ASVs were found among the core microbiota between forage and milk samples, they were not necessarily the most abundant in forage. Likewise, *P. pentosaceus* found at high relative abundance in the GLICI forage ration was not detected in the associated milk, whereas it was detected in milk samples from three other forage combinations showing lower relative abundance in forage (e.g., GL and GLC rations). Additionally, ASVs of the phylum Firmicutes were not identified in all forage rations and milk samples across the feeding combinations (Supplementary Figure S7). Surprisingly, 96% of the ASVs significantly enriched in milk samples from the H, GL, GLC, and GLICI feeding combinations when compared to each other (Supplementary Figures S4A,B) were members of those presumably transferred from forage (Supplementary Figure S7). Moreover, of the 39 ASVs significantly enriched in the GLICI milk compared with H, GL, and GLC counterparts regardless of the sampling period (Supplementary Figures S4A,B), 92% were part of the GLICI forage ration. Likewise, for the GLC type, 93% of the 40 ASVs enriched in raw milk were shared with silage. Lower proportions of 62% out of 39 ASVs and 63% out of 41 ASVs were observed in the GL and H typologies, respectively. Another noteworthy observation is the occurrence of ASVs of *Rhodococcus*, *A. guillouiae*, *Staphylococcus*, *Acinetobacter, Pseudoclavibacter*, an unidentified *Enterobacteriaceae*, and *Enterococcus* among those presumably transferred from forage.

## 4. Discussion

On dairy farms from which forage samples were collected, farmers implemented a mixed forage ration when applicable by adding corn silage to grass/legume silage, whether inoculated or not. We previously analyzed the microbiota of hay and ensiled forage types including grass/legume and corn silage both uninoculated or inoculated at harvest and found significant differences in their compositional and phylogenetic structure (Kennang Ouamba et al., 2022). In the current study, microbial communities composing these forage rations were grouped into three community types, broadly distinguishing H, GL, and GLICI from each other. Most of the GLC rations exhibited high similarity to GLICI. This suggests that in mixed forage rations involving grass/legume and corn silage, inoculating one or both types of feed would not lead to much difference in the resulting bacterial community composition compared to that of GLC. The observed phenomenon could be explained by the high prevalence and high relative abundance of *Acetobacteraceae* in corn silage, or *Leuconostocaceae* in uninoculated grass/legume silage as previously reported (Li and Nishino, 2011; Guan et al., 2018; Gagnon et al., 2020b). As discussed earlier (Kennang Ouamba et al., 2022), the genus *Weissella* was the main *Leuconostocaceae* in GL silage, where it was found to co-occur with undesirable taxa such as *Enterobacteriaceae*. This observation therefore emphasizes the advantages of the use of inoculants when ensiling grass/legume forage plants (Wang et al., 2006; Borreani et al., 2018). Further investigations involving more farm types will improve our understanding of the microbiological quality of mixed forage rations and refine our findings on their community types.

Following a cluster analysis, milk samples did not group according to forage rations, but rather revealed a strong effect of sampling period. Indeed, fall and spring milk samples were co-dominated by *Enterobacteriaceae* including ASVs of *Pseudomonas* and *Serratia*, and to a lesser extent by *Promicromonosporaceae* represented by *Cellulosimicrobium*. Moreover, these genera which have been associated with refrigerated raw milk (Rasolofo et al., 2010; Vithanage et al., 2016; Kennang Ouamba et al., 2020; Salgado et al., 2020) encompassed the most abundant ASVs differentially enriched across raw milk groups in both sampling periods, particularly in GLICI compared with GLC.

In the current study, the genus *Acinetobacter*, generally considered as part of the core microbiota of raw bovine milk (Quigley et al., 2013; Addis et al., 2016; Doyle et al., 2017a; Li et al., 2018), was not found among the 20 most relatively abundant genera. *Serratia liquefaciens* and *Pseudomonas* spp. have previously been reported as the predominant psychrotrophic bacteria that produce heat-resistant proteolytic and lipolytic enzymes with high spoilage potential (Machado et al., 2015; Salgado et al., 2020). In a concomitant study on forage microbiota (Kennang Ouamba et al., 2022), it was found that ASVs of *Pseudomonas* and *Serratia* were among the Proteobacteria that positively correlated with Firmicutes, and that members of these genera were generally more abundant in inoculated grass/legume silage compared with uninoculated counterparts. This suggests that in addition to warmer temperatures in the spring compared to fall, the use of the GLICI forage ration along with other farming practices implemented on farm probably contributed to higher loads of *Pseudomonas* and *Enterobacteriaceae* in the corresponding raw milk samples. Indeed, Li et al. (2018) showed that during cooler seasons, the growth of psychrotrophic bacteria such as *Pseudomonas* was favored, whereas *Firmicutes* and *Proteobacteria* correlated with higher temperatures. However, in this study, significantly higher loads of total bacteria, LAB, *Pseudomonas*, and *Enterobacteriaceae* were observed in GLICI raw milk samples compared with GL, as were *Pseudomonas* in GLICI compared with GLC, and total bacteria in GLC compared with GL in the spring compared to fall.

We observed irregular patterns of raw milk contamination by *Lactobacillales*, exhibiting only three ASVs as the core LAB from which two were assigned to the genus *Lactobacillus* and the last to *Lactococcus*. Similar results showing the sparseness of the core microbiota from 112 cow milk samples were reported by Li et al. (2018). These authors found that *Acinetobacter* and *Pseudomonas* were the sole genera shared by all milk samples they analyzed. Moreover, the meta-analysis performed by Bettera et al. (2023) clearly demonstrated the high prevalence of *Pseudomonas* and *Acinetobacter* in raw milk samples destined for cheesemaking. Regarding LAB, the same authors reported a high prevalence of the genus *Lactococcus* (~99%). Kable et al. (2016) reported a more diverse core microbiota encompassing 29 taxa at the genus level (from 899 raw milk samples), among which the LAB community was represented by unidentified *Aerococcaceae*, *Enterococcus*, and *Streptococcus*. Recently, Parente et al. (2020) used the FoodMicrobionet database to analyze the results from five studies that examined a total of 199 bulk tank milk samples from five different regions around the world, and found that the genera *Pseudomonas*, *Streptococcus*, *Lactococcus*, and *Acinetobacter* showed the greatest prevalence rates of ~98, ~97, ~93, ~93%, respectively. Bettera et al. (2023) used an updated version of the FoodMicrobionet database and obtained similar results, showing that LAB were not the most abundant bacterial groups in the raw milk microbiota. They found that species belonging to *Lactobacillus* and *Lacticaseibacillus* were present in lower relative abundance in comparison with *Lactococcus* and *Streptococcus*. Using a culture-dependent approach, Gagnon et al. (2020b) analyzed 1,239 LAB isolates from 48 bulk tank milk samples and found that *Lcb. casei/paracasei*, *P. pentosaceus*, *Weissella paramesenteroides* or *Weissella thailandensis*, and *Lactococcus lactis* were the most prevalent with 60, 42, 40, and 30% prevalence rates, respectively. Moreover, these authors revealed that despite the substantial enrichment of 35% in *Lactobacillaceae* in the microbiota of inoculated grass/legume silage compared with uninoculated counterparts, the associated milk samples exhibited similar LAB profiles. These findings support the hypothesis that there is not a clearly defined pattern of raw milk contamination on dairy farms, particularly for LAB.

The genera *Streptococcus*, *Staphylococcus*, *Corynebacterium*, *Enterococcus*, *Acinetobacter*, as well as members of *Enterobacteriaceae* have been commonly identified as mastitis causing agents (Dufour et al., 2012; Falentin et al., 2016; Gonçalves et al., 2016; Derakhshani et al., 2018a). Regarding these taxa, our findings corroborate those of Rodrigues et al. (2017) who reported positive correlations with milk somatic cell counts. The genera *Jeotgalicoccus*, *Bifidobacterium*, and *Solibacillus* were found among the most abundant taxa of the bovine teat microbiota (Falentin et al., 2016; Rault et al., 2020), while *Brevundimonas* was identified as a dominant taxon in clinical mastitis samples (Kuehn et al., 2013). In the current study, except for *Enterobacteriaceae*, none of the ASVs encompassing these taxa were found to be differentially abundant among milk samples associated with the five forage combinations.

Our results show that silage-based forage rations, particularly GLC and GLICI, share more ASVs with raw milk produced on corresponding farms compared to that observed in the milk from cows fed a H ration. Among the 113 presumably transferred ASVs, Proteobacteria were by far the most represented compared to Firmicutes and Actinobacteria, each at 65, 23, and 12%, respectively. Rather than observing a significant enrichment of *Lactobacillaceae* in milk samples from the GLICI forage type as they dominated the microbiota of the corresponding forage ration, ASVs assigned to *Enterobacteriaceae* (mainly *Serratia*, unidentified *Enterobacteriaceae*, *Yersinia*, and *Hafnia-Obesumbacterium*), *Pseudomonadaceae* (*Pseudomonas*), *Promicromonosporaceae* (*Cellulosimicrobium*), and *Aeromonadaceae* (*Aeromonas*) were listed among the differentially abundant taxa and were the most represented. Interestingly, 92% of ASVs enriched in the GLICI milk, among which all those cited above, were identified in the microbiota of the associated forage ration, as were 93% of those enriched in the GLC milk. However, these proportions were considerably reduced in the ration involving a single forage type (H or GL). These findings clearly demonstrate that although bacteria from forage may represent a low proportion of the associated raw milk microbiota, they may be the main taxa distinguishing between milk from different feeding combinations. Our results show that the mixture of grass/legume and corn silage significantly impacts the raw milk microbiota compared with a single forage-based ration. Considering the case of GLICI versus GLC raw milk samples, it appears that differences in their microbial communities were mostly driven by greater relative abundance of Proteobacteria in the GLICI forage type. However, this is not specific to GLICI milk as similar observations can be made when the same comparison is performed between other feeding combinations. Therefore, based on the current study, it is difficult to provide reliable explanation on a direct influence of silage inoculants on raw milk microbiota upon milking.

However, the significance of other sources of milk contamination may explain the observed low proportions of the shared ASVs relative to those uniquely occurring in the forage types or milk samples from the same feeding combination. Sources of microorganisms include the bedding material, feces, cow skin, water, humans, milking machines and pipelines, bulk tank, air, pasture, and other feed components (Gleeson et al., 2013; Quigley et al., 2013; Derakhshani et al., 2018b; Gagnon et al., 2020a; Ouamba et al., 2022). These observations suggest a lack of correlation between taxa abundance in forage and their abundance in milk. Supporting this hypothesis, Driehuis (2013) reported in their review dealing with the impact of silage on the quality of dairy foods that raw milk contamination by aerial spores from silage or by direct contact of raw milk with silage are negligeable when milking hygiene is properly applied. However, higher levels of milk contamination with spores has been correlated with higher abundance of spore-forming bacteria in silage (Abeni et al., 2021). Bacteria from silage take indirect milk contamination routes, possibly involving a sporadic transfer of silage onto the bedding or directly to the cow skin (of which the teat surface is cleaned before milking), improper human handling, or via feces that can contaminate the bedding and the teat surface. Indeed, spore-forming bacteria from silage were found to withstand harsh conditions along the cow gastrointestinal tract and subsequently end up in the feces (Pahlow et al., 2003; Driehuis et al., 2016). Although still under debate, it should be considered that silage bacteria may translocate via the entero-mammary pathway previously described (Rodríguez, 2014; Addis et al., 2016; Oikonomou et al., 2020). On a dairy farm, the interconnections among the microbial sources, which by themselves can be selective habitats, might explain why a clear pattern of milk contamination by silage bacteria was not in evidence in this study.

## 5. Conclusion

In summary, the microbiota of forage types analyzed in this study were grouped into three community types broadly distinguishing between H, GL, and GLICI samples, GLC showing high similarity with GLICI. However, a subsequent classification of microbial communities in milk associated with the forage ration combinations was not observed. The effect of forage ration combinations on the milk microbiota appeared more substantial in spring, as significantly higher loads of LAB, *Pseudomonas*, *Enterobacteriaceae*, and total bacteria were observed in GLICI compared with milk samples associated with other feeding combinations. This study was carried out using freshly produced bulk tank milk, for which we demonstrated irregular patterns of contamination on farm. Bacteria from forage rations encompassing H, GL, GLC, and GLICI may account for up to 31% of the microbial community in the corresponding milk. Trends of direct contamination of milk by forage bacteria were not evidenced for any of the 113 ASVs presumably transferred from forage to milk. Although significant differences were observed between GLICI and GLC milk samples, they were driven more by *Enterobacteriaceae* and other Proteobacteria, rather than by LAB communities. Drawing reliable conclusions on the influence of silage inoculants on the raw milk microbial community is therefore challenging. This study however provides new insights into the microbial structure of forage rations fed to cows and associated raw milk from commercial dairy farms. The identity of bacterial species that are likely transferred from forage rations to raw milk is provided, demonstrating the relevance of cow forage rations as sources of bacteria that contaminate raw milk on farms. Additional milk samples taken at the end of the transport chain from dairy farms to processing plants might reveal further effects of forage combinations on raw milk microbiota. Further investigation involving more farm types and the integration of metagenomics and metabolomics would be needed to better understand the impact of cow feeding with inoculated silage on milk quality and processability.

## Data availability statement

The datasets presented in this study can be found in online repositories. The names of the repository/repositories and accession number(s) can be found at: https://www.ncbi.nlm.nih.gov/, PRJNA938929.

## Author contributions

DR, GL, and PC: conceptualization, validation, and funding acquisition. AO and MG: methodology. AO and TV: software. AO: investigation, data curation, writing – original draft preparation, and visualization. DR: resources, supervision, and project administration. AO, MG, DR, GL, and PC: writing – review and editing. All authors have read and agreed to the published version of the manuscript.
