# Supplementary material for: Phylogenetic variation in raw cow milk microbiota and the impact of forage combinations and use of silage inoculants
Source: Front Microbiol. 2023 Nov 7;14:1175663. doi: 10.3389/fmicb.2023.1175663 (PMC10661925; doi:10.3389/fmicb.2023.1175663)
Supplement: Supplementary file 1 [file Data_Sheet_1.pdf]

Supplementary Material

Phylogenetic variation in raw cow milk microbiota and the impact of forage combinations and use of silage inoculants

Alexandre J. K. Ouamba, Mérilie Gagnon, Thibault Varin, P. Yvan Chouinard, Gisèle LaPointe, Denis Roy*

*** Correspondence:** Corresponding Author: Denis.Roy@fsaa.ulaval.ca

1 Supplementary Figures and Tables

1.1 Supplementary Figures

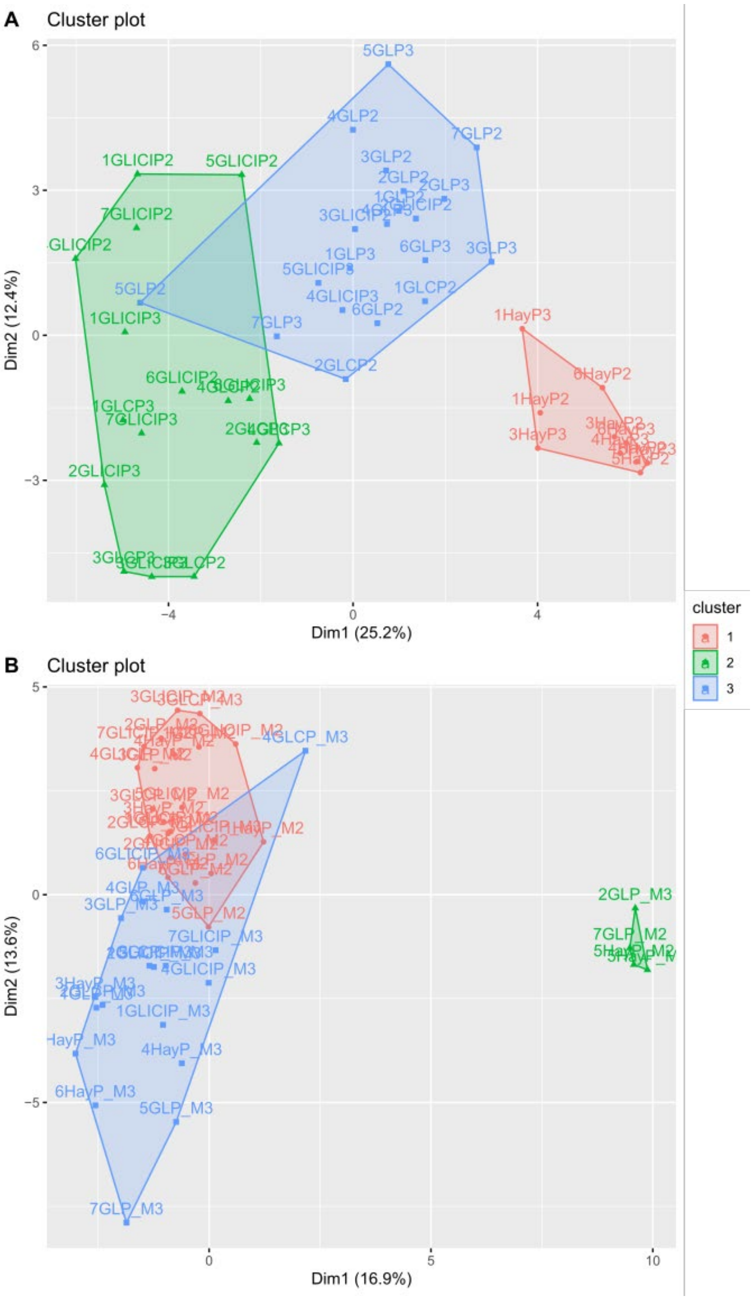

Supplementary Figure S1. Principal component analysis showing the separation of forage ration bacterial communities (A) and associated raw milk samples (B) in three distinct clusters. Clustering was computed using the portioning around medoids algorithm.

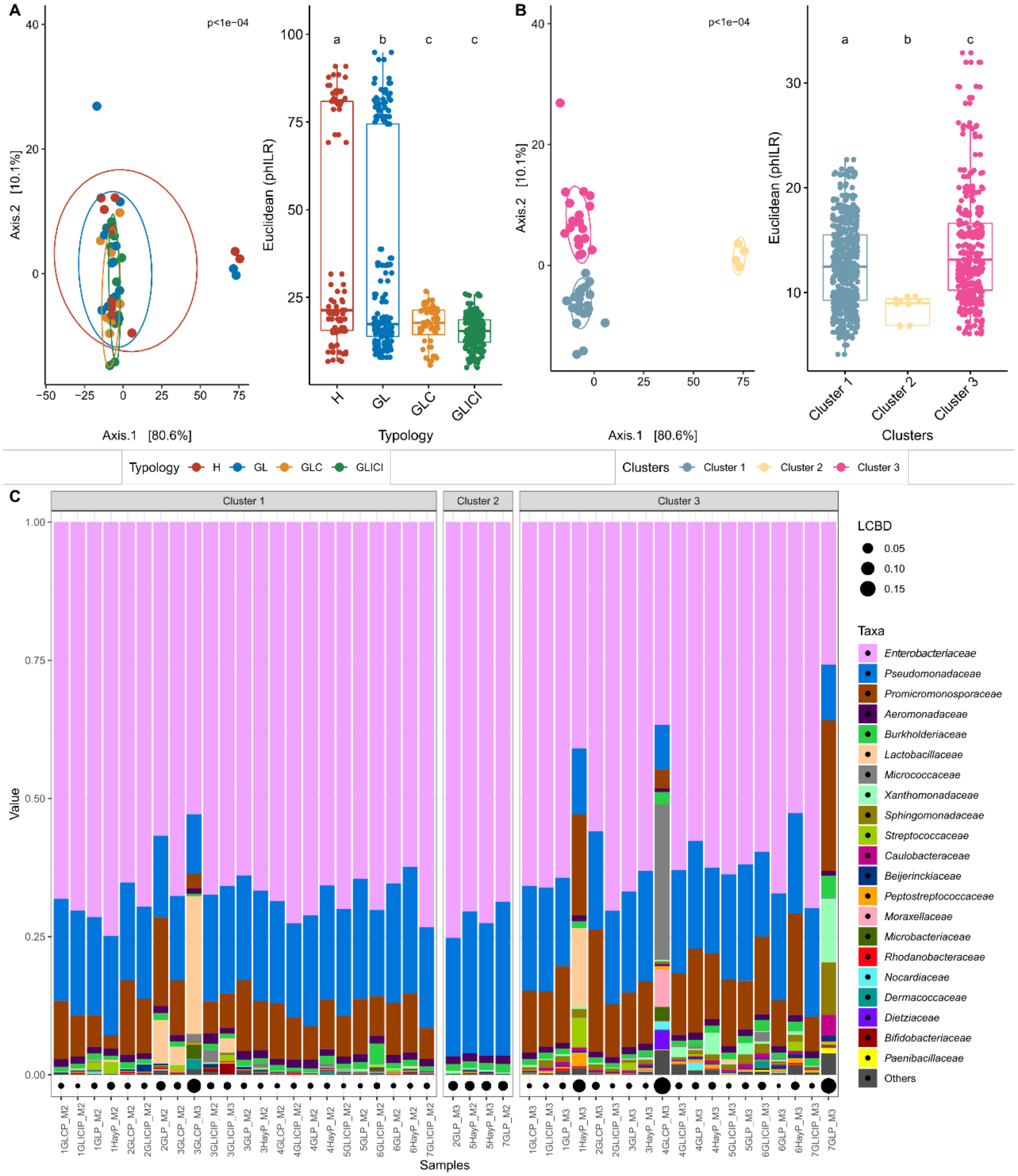

Supplementary Figure S2. Cluster analysis of milk samples. Principal component analysis based on PhILR transformed data (left) with corresponding post-hoc tests (right) for milk samples (A) and derived clusters (B). Milk sample types or clusters associated with different letters are significantly different based on Wilcoxon rank sum test with FDR correction. (C) Relative abundance of the 21 most abundant families occurring in the defined clusters. Local contribution to beta diversity values denotes the indices of sample local contribution to the observed beta-diversity between groups. Values are proportional to sample contribution to beta-diversity.

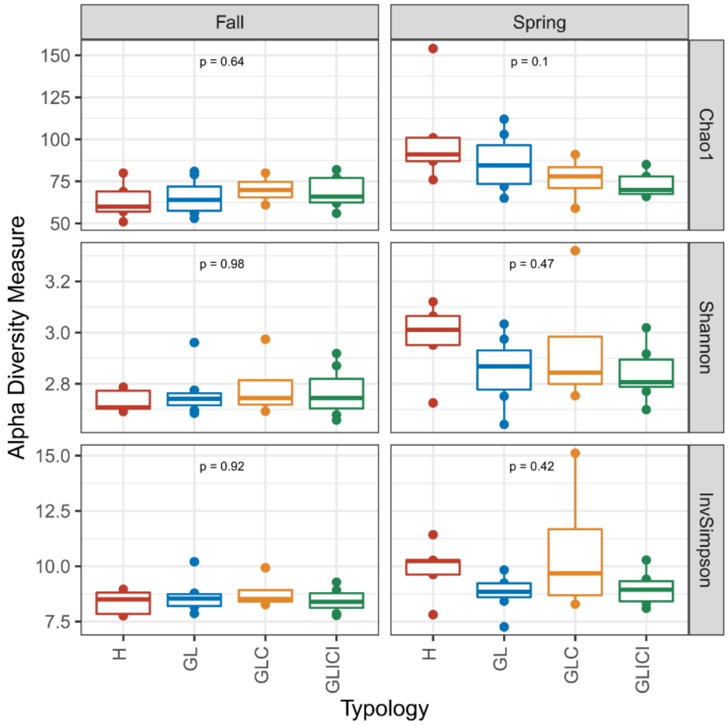

Supplementary Figure S3. Alpha-diversity of milk bacterial communities. p values indicate the significance of the Kruskal-Wallis test performed to compare milk samples from distinct feeding combinations.

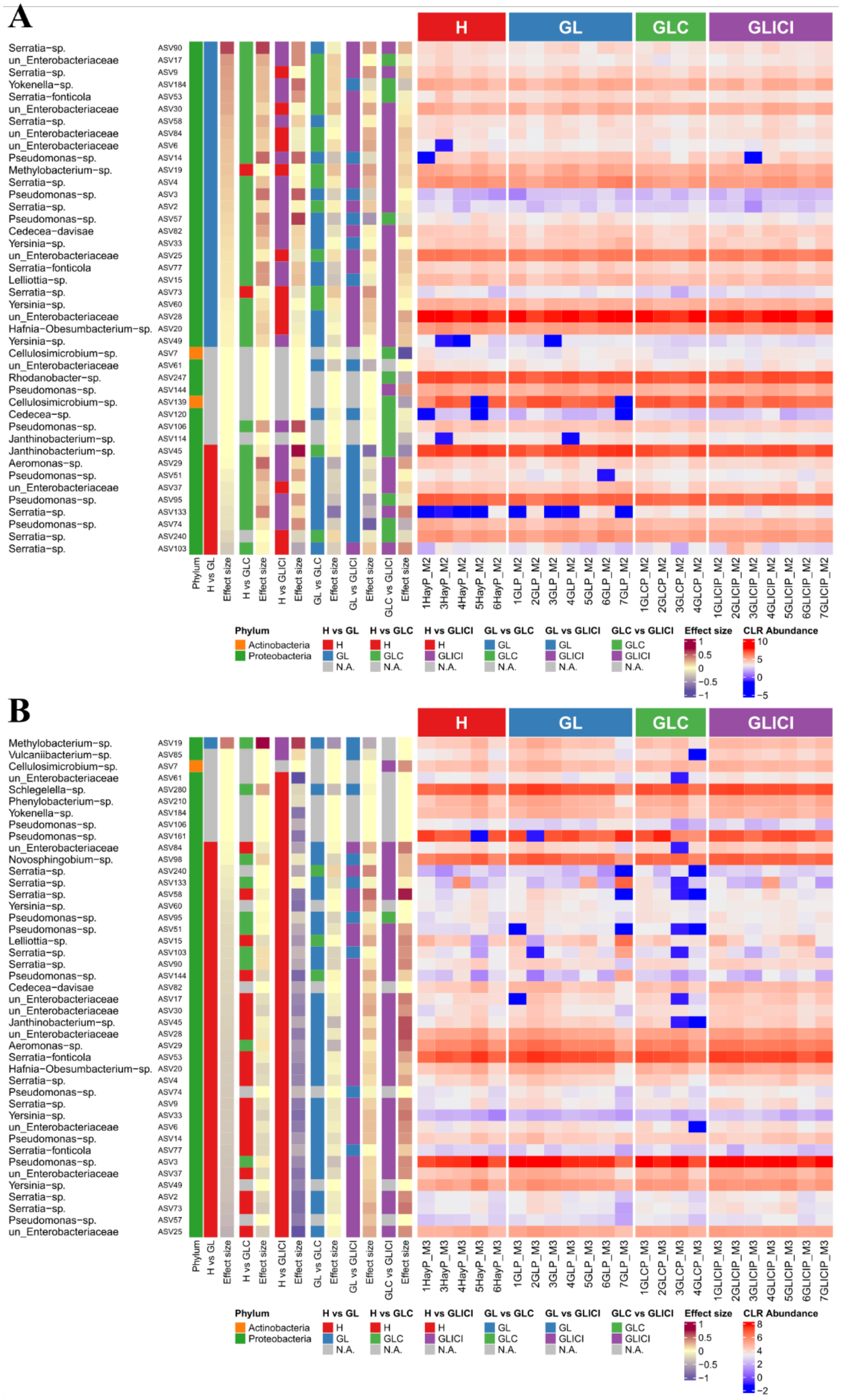

Supplementary Figure S4. Differentially abundant taxa among milk samples. Differential abundance was performed using the ADEx2 algorithm on microbial community data from milk samples collected in the fall (A) and the spring (B). The heatmap illustrates the distribution and

abundance of differentially abundant taxa across feeding types. Corresponding paired group comparisons, effect sizes, and taxonomic classification (phylum level) are displayed as heatmap annotation on the left.

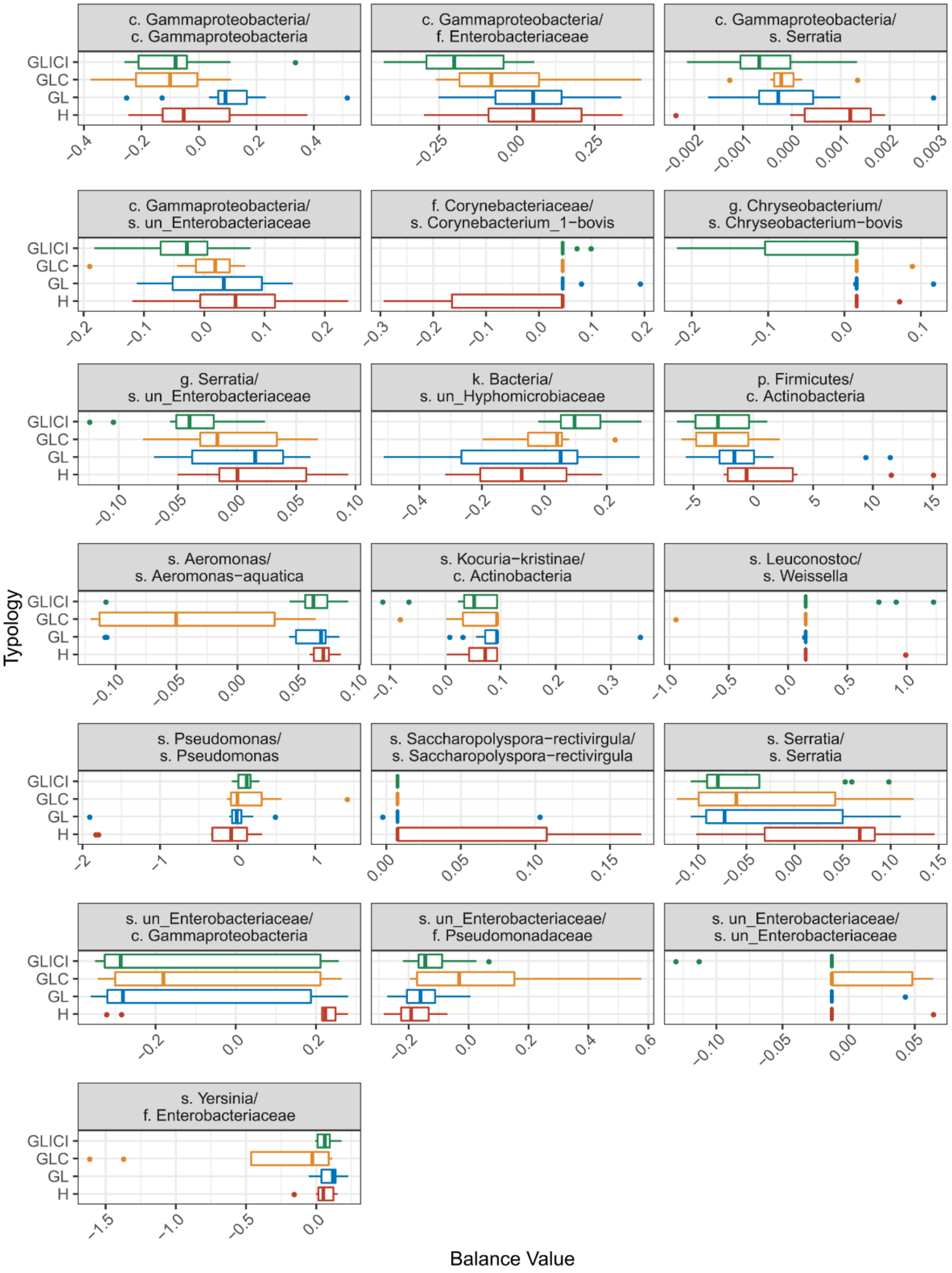

Supplementary Figure S5. Phylogenetic balances separating milk microbiota by forage type. For example, the balance of the phylum Firmicutes relative to the class Actinobacteria discriminates H from GLICI and GLC milk samples. Firmicutes are represented by *Clostridium disporicum*,

Clostridium sp., *Paeniclostridium* sp., *Coprococcus* sp., *Romboutsia sedimentorum*, *Romboutsia* sp., *Veillonella dispar*, and an unclassified *Peptostreptococcaceae* while Actinobacteria include *Bifidobacterium* spp., *Cellulosimicrobium* spp., *Kocuria* spp., *Corynebacterium* spp., *Saccharopolyspora rectivirgula*, and more.

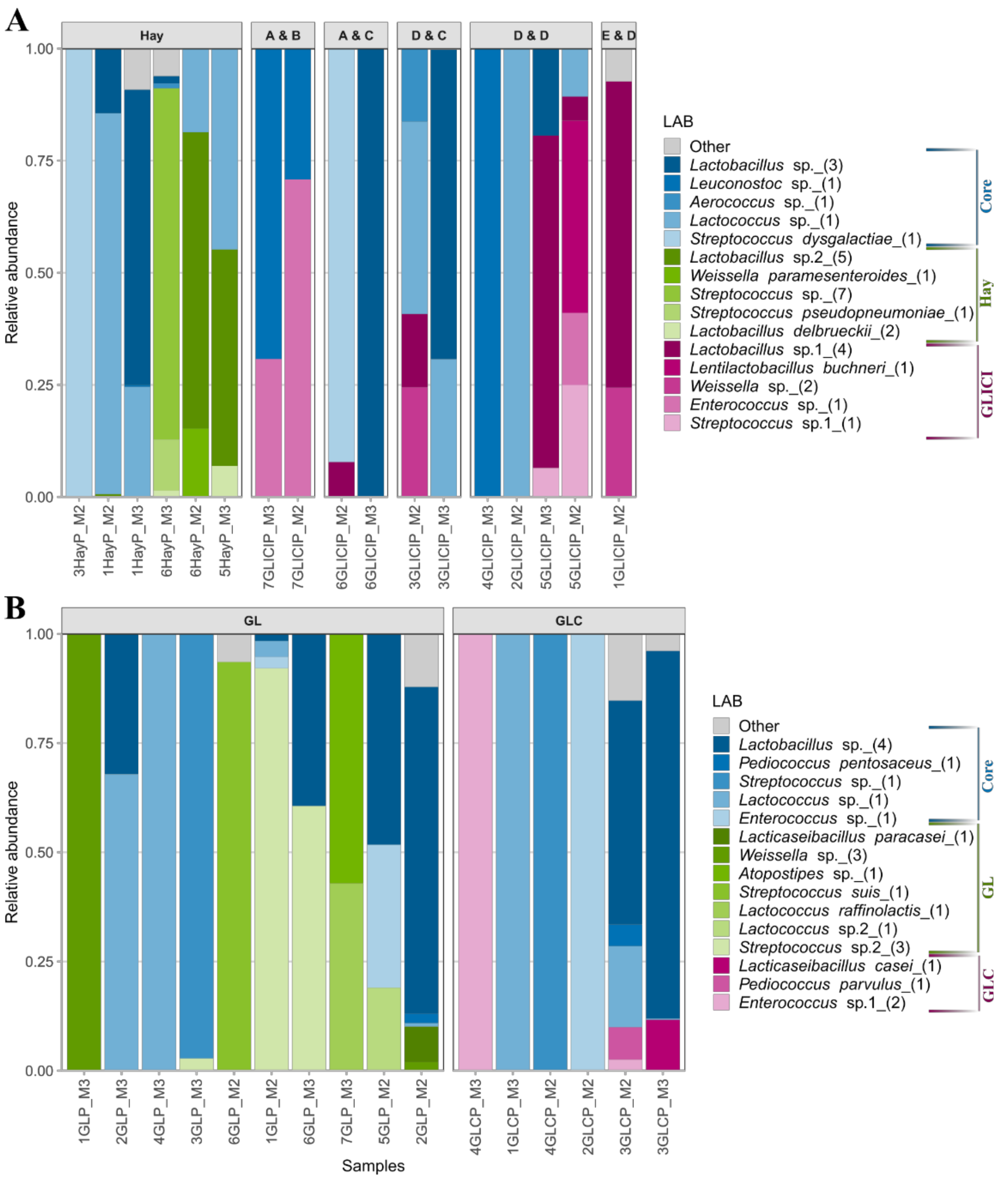

Supplementary Figure S6. Distribution and proportion of LAB communities among milk groups. Proportion of the core and unique LAB phylotypes among GLICI and H (A) or among GLC and GL (B) milk samples. Milk samples from inoculated forage ration combinations are separated according to the inoculant brands used for the grass or legume (first letter) and corn (second letter) silage. Accordingly, “A” = 11G22, “B” = 11C33, “C” = 11CFT, “D” = Biotal Buchneri 500, and “E” = Biotal Supersile. Taxa are coloured according to whether they are unique to one or the other group (GLICI or H, GLC or GL) or whether they belong to both (core).

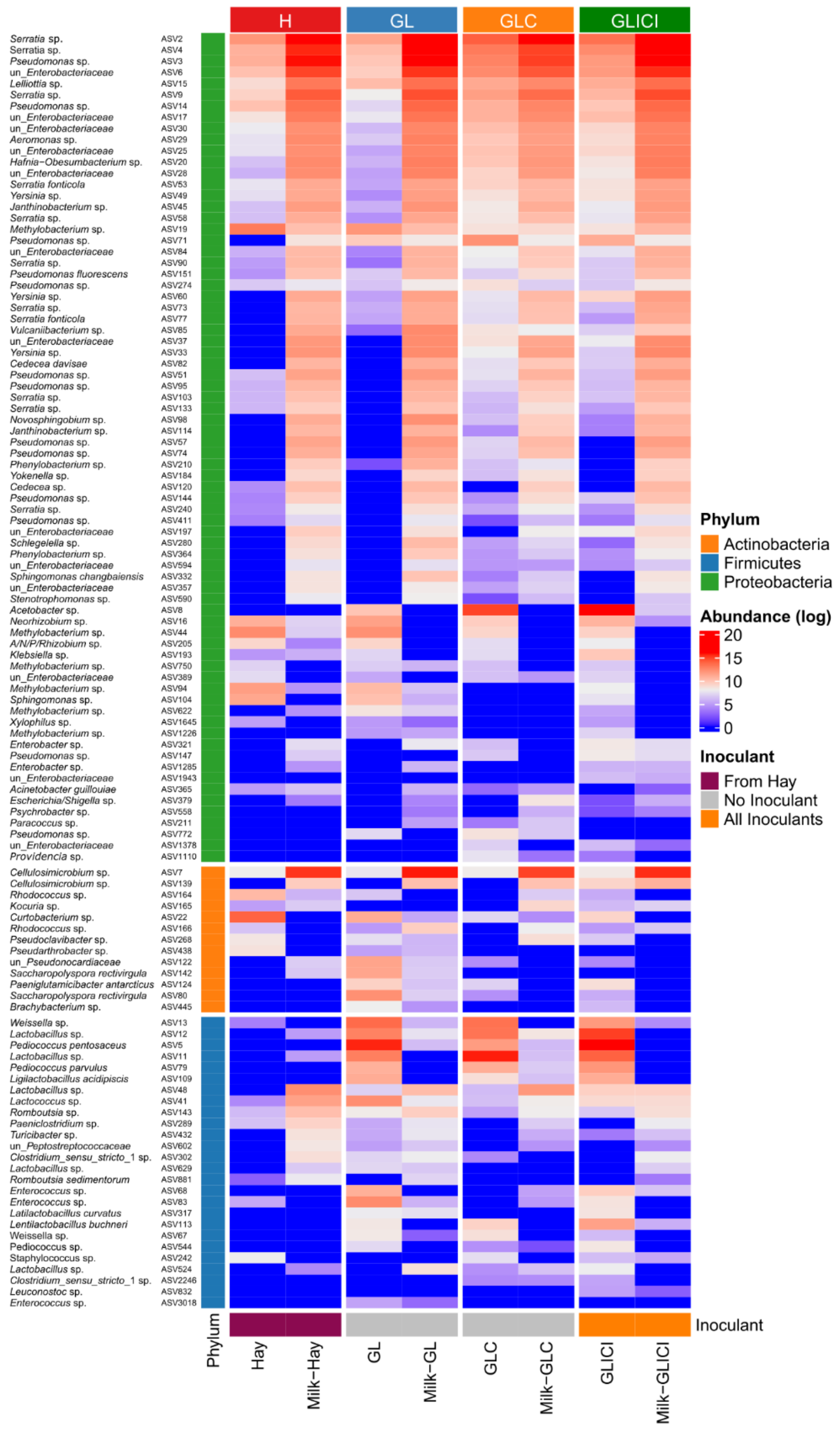

Supplementary Figure S7. Distribution, prevalence, and abundance of shared phylotypes between forage types and milk samples.
